# Fertilization and Global Warming Impact on Paddy CH_4_ Emissions

**DOI:** 10.3390/ijerph20064680

**Published:** 2023-03-07

**Authors:** Pengfu Hou, Xuzhe Deng, Jing Wang, Lixiang Xue, Yushu Zhang, Tingting Xu, Lihong Xue, Linzhang Yang

**Affiliations:** 1 Key Laboratory of Agro-Environment in Downstream of Yangze Plain, Ministry of Agriculture and Rural Affairs of China, Jiangsu Academy of Agricultural Sciences, Nanjing 210014, China; 2 Co-Innovation Center for Sustainable Forestry in Southern China, College of Forestry, Nanjing Forestry University, Nanjing 210037, China; 3 Fujian Key Laboratory of Plant Nutrition and Fertilizer, Institute of Soil and Fertilizer, Fujian Academy of Agricultural Sciences, Fuzhou 350013, China; 4 College of Resources and Environmental Sciences, Nanjing Agricultural University, Nanjing 210095, China

**Keywords:** warming, fertilization, CH_4_ emissions, rice yield, soil fertility, soil microorganisms

## Abstract

Introduction: This study aimed to assess the influence of experimental warming and fertilization on rice yield and paddy methane emissions. Methods: A free-air temperature increase system was used for the experimental warming treatment (ET), while the control treatment used ambient temperature (AC). Each treatment contained two fertilization strategies, (i) normal fertilization with N, P and K fertilizers (CN) and (ii) without N fertilizer input (CK). Results: The yield was remarkably dictated by fertilization (*p* < 0.01), but not warming. Its value with CN treatment increased by 76.24% compared to CK. Also, the interactive effect of warming and fertilization on CH_4_ emissions was insignificant. The seasonal emissions from warming increased by 36.93% compared to AC, while the values under CN treatment increased by 79.92% compared to CK. Accordingly, the ET-CN treatment obtained the highest CH_4_ emissions (178.08 kg ha^−1^), notably higher than the other treatments. Also, the results showed that soil fertility is the main driver affecting CH_4_ emissions rather than soil microorganisms. Conclusions: Fertilization aggravates the increasing effect of warming on paddy methane emissions. It is a daunting task to optimize fertilization to ensure yield and reduce methane emissions amid global warming.

## 1. Introduction

Greenhouse gas (GHG) emitted from agro-ecosystems is one of the key culprits of climate warming [1]. Paddy methane (CH_4_) emissions are the biggest contributor, accounting for ~48% of cropland GHG emissions [2]. However, everything is reciprocal. The increased atmosphere temperature may in turn affect CH_4_ production and emission in paddies. Liu et al. declared that warming (~1.5 °C) enhanced paddy methane emissions by 23.4% by synthesizing global data [3]. This suggests that attention should also be paid to the feedback of climate warming on CH_4_ emissions in rice fields.

According to the IPCC, the surface temperature in the period 2010 to 2019 caused by human activity increased by ~1.07 °C compared to the period 1850 to 1900 [4]. Research in a rice-wheat cropping field observed an accelerating effect of warming to paddy CH_4_ emissions [5]. It also found that the results were closely related to the abundance of methanogen. However, this trend is not static, e.g., Wang et al. found that the treatment effect of induced warming on CH_4_ emitting from double-cropping paddies was negligible [6]. Using a multi-site experiment, Qian et al. found that the response of paddy CH_4_ emissions to warming was closely related to the surrounding meteorology [7]. Warming only significantly promoted paddy CH_4_ emissions at medium temperatures (~27 °C) due to the lower plant biomass and/or higher methanogens copies, while it showed insignificant effect at lower (~21 °C) or higher temperatures (~31 °C). The above results imply that the response of CH_4_ emissions from paddies to warming may not be consistent, and closely related to the planting area/system (environmental temperature).

It is well known that CH_4_ emission is the residual product of methanogenic metabolites utilized by methanotrophs [8,9]. As mentioned above, the varieties in soil organic carbon (SOC), plant biomass and the abundance of microorganisms associated with methane emissions may affect paddy methane emissions under warming [3,7]. Specifically, changes in crop growth are accompanied by changes in root oxygen secretion [10]. Furthermore, changes of temperature may affect soil carbon turnover and soil physicochemical properties [11,12,13]. Also, changes in environmental factors can affect the abundance of methanogens and methanotrophs in paddy soils [5,7,14]. These may affect the processes of methane production and oxidation, thus affecting CH_4_ emissions.

Notably, one factor change may cause the linkage effect of other factors; e.g., increased root oxygen secretion with crop growth can accelerate soil mineralization and promote the copies of methanogens and methanotrophs [10,15]; the response of soil carbon turnover to temperature is also closely related to microorganisms [12]. Combined with the above analysis, it is not clear which indicator is the key factor affecting methane emissions, especially as the response of paddy CH_4_ emission to warming varies with background temperature. Additionally, a noteworthy problem is that all the results of methane emissions in response to warming do not take into account the effects of fertilization. Chemical nitrogen (N) fertilizer is a vital source to improve cereal yield and ensure food security. Numerous studies have shown that chemical N-fertilizer may affect methane emissions in paddies by altering crop growth, soil fertility and microbial abundance [16,17]. In this context, it is unknown whether the response of methane emitting from paddy fields to warming is affected by fertilization.

Here, we hypothesized that fertilization could aggravate the increasing effect of warming on paddy methane emissions by promoting crop growth, accelerating SOC turnover and changing soil physicochemical properties as well as microbial abundance. To verify the hypothesis, we undertook a field trial to assess the influence of experimental warming and fertilization on rice yield and paddy methane emissions. The correlation between CH_4_ emission performance and rice yield, soil fertility, as well as the copies of functional microbial genes in relation to CH_4_ production (*mcr*A, the highly conserved and specific gene for methanogens [18]) and oxidation (*pmo*A, the highly conserved gene used for a functional marker of methanotrophs [19]) was also analyzed. We expect this study can provide a reference for the mitigation of paddy CH_4_ emissions amid global warming.

## 2. Materials and Methods

### 2.1. Experiment Site

The employed rice-wheat cropping field was located in Fuzhuang Village, Nanjing City, China (119.07° E, 32.00° N), a region with annual paddy-upland rotation cropping regime. The annual mean precipitation and temperature were 1106.5 mm and 15.4 °C, respectively. The basic properties of the experimental soil were: pH, 5.93, soil organic matter (SOM), 29.22 g kg^−1^ and total nitrogen (TN), 1.95 g kg^−1^.

### 2.2. Experimental Design

The field experiment was initiated in June 2018 and continued from that rice season. The data collection for the present study was conducted in 2020. Treatments with experimental warming (ET) and without warming (AC, the control group with natural ambient conditions) were selected as the main treatment with three replicates. All plots were subjected to the same fertilization treatment (CN). In the three ET plots (42 m^2^ per plot), the free-air temperature increase equipment (FATE) was established before planting rice in 2018 and continued running from that rice season. The effective warming area for the FATE was 3.6 m^2^ for each plot. Besides, similar ‘dummy’ equipment was installed in the three AC plots to mimic the shading effect of the heater. Overall, mean diurnal canopy temperature under ET increased by 0.7~1.3 °C between the three rice growing seasons, while diurnal soil temperature increased 0.7~1.1 °C. To clarify the mutual impact of warming and fertilization to paddy methane emission, sub-micro-plot (0.25 m^2^) with no N-fertilizer input (CK) was set up in each treatment plot.

The fertilization operation in CN treatments was kept the same. The urea-N (270 kg N ha^−1^) was spread 3 times at basal (35%), tillering (30%) and panicle initiation stages (35%), whilst the phosphorus (calcium superphosphate, 108 kg P_2_O_5_ ha^−1^) and potassium (potassium chloride, 216 kg K_2_O ha^−1^) fertilizers were thoroughly broadcast before transplanting. Except that no N-fertilizer was applied, other farming operations in CK trial were consistent with those from CN trial. The rice variety trialed was Nanjing 5055, a conventional japonica rice type. The seedling was manually transplanted in mid-June with unified spatial distribution (30 cm × 14 cm). For the water regime, following a conventional local water management strategy, except for mid-season drainage and drainage for rice ripening, paddies were irrigated with continuous flooding during other periods of rice growth. The farming operations in wheat season are given by Ma et al. [20].

### 2.3. Sampling and Measurement

At maturity, 0.5 m^2^ plant samples in CN plots and all CK plots (0.25 m^2^) with/without warming were harvested, air-dried and threshed to measure the rice yield. The static-chamber method was used for collecting the gas samples throughout the rice growing season from 8:00 to 10:00 AM. Gas samples were collected on the 1st, 3rd, 5th, 8th day after each fertilization (basal, tillering and panicle fertilization) every 2 days during the middle drainage management period (the 1st, 3rd and 5th day after drainage) and every 7 to 10 days during other periods. Due to the dominating role of CH_4_ emissions in greenhouse gas emissions in paddies, we only focused on the effects of warming and fertilization on paddy CH_4_ emissions in the present study. The gas samples were collected at 10 min intervals for a total of 30 min (0-, 10-, 20-, 30-min). After that, the gas concentration of CH_4_ was measured by Agilent 7890B (Agilent Technologies, Santa Clara, CA, USA) and the CH_4_ concentration inside the chamber as a function of time was used to calculate the CH_4_ emission rate in unit sampling time. Only the measurements that had *r^2^* > 0.90 were accepted. The seasonal cumulative CH_4_ emissions were computed by adding the emissions between two adjacent measurement intervals. Then, the methane emission intensity was computed according to Mosier et al. [21].

Soil samples of plough layer (0~20 cm) were taken from five random points in each plot after rice was harvested. Samples were air-dried to measure the soil pH, organic matter (SOM), total nitrogen (TN), labile organic carbon (LOC) as well as alkaline-hydrolyzable N (AN) and the C/N ratio. The soil pH was measured by mixing the deionized water with soil sample at a ratio of 2.5; the contents of SOM, LOC, TN and AN were measured using the potassium dichromate volumetric and external heating method, the potassium permanganate oxidation method, the Kjeldahl method and the alkaline hydrolysis diffusion method, respectively [22,23]. Also, fresh soil samples during the peak emitting stage of CH_4_ flux were also taken to analyze the copies of *mcr*A and *pmo*A genes with the qPCR. The primers (mlas/mcrA-rev, A189F/Mb661R) and operating procedure were provided by Hou et al. [17].

### 2.4. Statistical Analysis

Two-way ANOVA was employed to analyze the effects of experimental warming and fertilization on rice yield, CH_4_ emission, soil physicochemical properties and functional microbial genes. The significant differences of the variables were determined using the LSD method. SPSS 16.0 and OriginPro 2021 software were employed for the data analysis and mapping, respectively. *p* < 0.05 was considered as the threshold value for statistical significance. Also, SIMCA 13.0 was selected to analyze the relative importance of soil physicochemical properties and functional microbial genes to the rice yield and methane emissions (cut-off value with 1.0).

## 3. Results

### 3.1. CH_4_ Emissions

**Figure 1:**
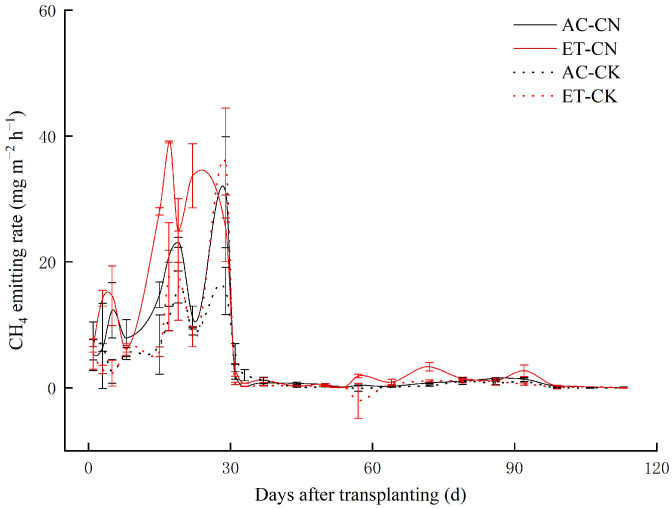
Paddy CH_4_ emitting rate (means ± se) as affected by temperature and fertilization. AC, ambient control; ET, experimental warming; CN, fertilization treatment; CK, no N-fertilizer input treatment.

Figure 1 shows that the CH_4_ emissions in paddy fields were mainly concentrated in the period from transplanting to midseason aeration. After that, the CH_4_ emission rates decreased rapidly and remained low. Under the same fertilization, the CH_4_ emission rates subjected to experimental warming were obviously higher than those under the ambient control treatment at most monitoring times. Meanwhile, the emitting rates in CN treatment were also distinctly higher than that in CK treatment. As a response, warming and fertilization both significantly enhanced the average CH_4_ emitting rate (*p* < 0.05 or 0.01), while their interactive effect was insignificant (Table 1). The ET-CN treatment achieved the highest average CH_4_ emitting rate (8.24 mg m^−2^ h^−1^); the value was notably higher than the other treatments.

### 3.2. Grain Yield and CH_4_ Emissions

Consistent with the methane emitting rate, the seasonal CH_4_ emissions were notably dictated by fertilization and warming (*p* < 0.05 or 0.01) (Table 1). The seasonal CH_4_ emissions from warming (ET) increased by 36.93% in comparison to ambient control (AC). Meanwhile, in comparison with CK the values under CN treatment increased by 79.92%. According to this, the ET-CN treatment obtained the highest CH_4_ emissions (178.08 kg ha^−1^), notably higher than the other treatments.

**Figure 2:**
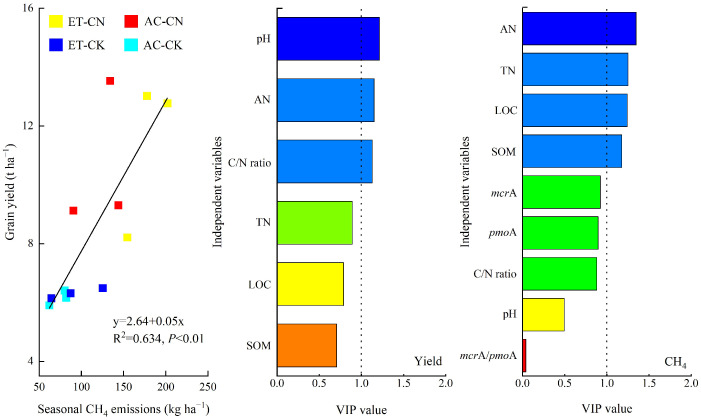
Correlation between cereal yield and paddy CH_4_ emissions and the variable importance for the projection (VIP) values of independent variable effects on rice yield and methane emission (cut-off value with 1.0).

As shown in Table 1, the rice yields were only notably dictated by fertilization (*p* < 0.01). Their value with CN treatment increased by 76.24% relative to the CK. Due to the notable increase effect of fertilization on CH_4_ emission and yield, the grain yield was positively correlated with seasonal methane emissions (*p* < 0.01) (Figure 2). Accordingly, fertilization treatments (CN) achieved similar values of CH_4_ emission intensity with no-N fertilizer control (CK). Also, warming only showed a tendency to increase the CH_4_ emission intensity (*p* < 0.1).

### 3.3. Soil Fertility

Table 2 shows that the interactive effects of warming and fertilization on soil physicochemical properties were not significant. Compared with the no-N fertilizer control (5.98), the soil pH with fertilization decreased to 5.70 (*p <* 0.05). Also, fertilization clearly increased the labile organic carbon (LOC) and alkaline hydrolyzable N (AN) contents by 12.50% and 8.69%, respectively. The soil organic matter (SOM) and total N (TN) contents were also increased by 3.97% and 6.11%, respectively, although the difference among the treatments was insignificant. Additionally, fertilization tended to reduce the soil C/N ratio in comparison to the CK control.

Table 2 also indicates that, compared with ambient control (AC), warming (ET) had a tendency to increase the soil pH and LOC content by 3.81% and 10.97%, respectively. In addition, warming increased the SOM, TN and AN contents by 7.32%, 8.11% and 6.59%, respectively, although the difference among the treatments was insignificant. The C/N ratio between ET and AC remained similar. The variable importance for the projection (VIP) analysis indicated that soil pH, AN as well as the C/N ratio were the main drivers in response to grain yield (Figure 2), while soil AN, TN, LOC and SOM could affect paddy CH_4_ emissions.

### 3.4. Abundance of Soil Fuctional Microbial Genes

**Figure 3:**
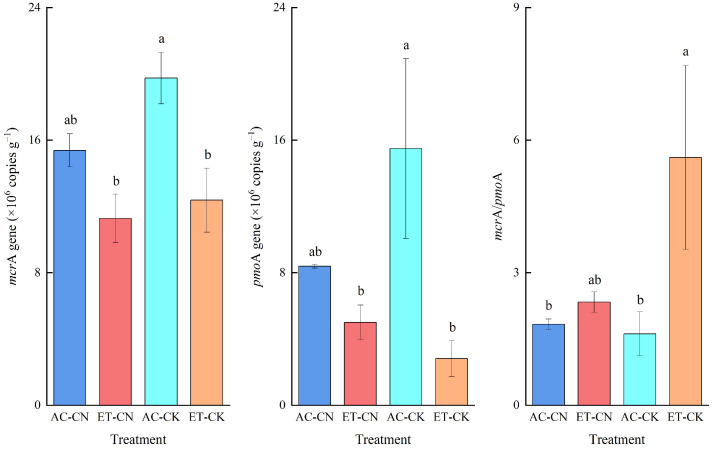
The copies of *mcr*A, *pmo*A genes and *mcr*A/*pmo*A ratio as affected by temperature and fertilization. Different letter on the bars means the significant difference with confidence level set at 0.05.

Table 3 shows that the copy numbers of *mcr*A and *pmo*A genes were notably dictated by warming, but not from fertilization. In comparison to the AC, ET remarkably decreased both the copies of *mcr*A and *pmo*A genes (*p* < 0.05 or 0.01). Specifically, the AC-CK trial showed the highest values of *mcr*A and *pmo*A genes and notably higher than the ET-CN and ET-CK treatments (Figure 3). Due to the different variation ranges of *mcr*A and *pmo*A genes, the *mcr*A/*pmo*A from the ET-CK trial was remarkably higher than those from the AC-CN and AC-CK treatments with the highest value. Surprisingly, the VIP analysis indicated that the copies of *mcr*A and *pmo*A genes and *mcr*A/*pmo*A all showed no significant effect on paddy CH_4_ emissions (Figure 2).

## 4. Discussion

### 4.1. Rice Yield

N fertilizer plays a critical role in enhancing global crop production [24]. Here, the present results confirmed this phenomenon (Table 1). The average rice yield of CN treatment increased by 76.24% in comparison to that achieved from the no-N fertilizer control. Importantly, we found that the grain yields between warming and ambient control remained similar. Wang et al. also found that the impact of warming on rice yield in double-cropping paddies was negligible [6]. Conversely, Wang et al. pointed out that warming reduced the rice yield by 5.4~20.3% in both monitoring years in a rice-wheat rotation [25]. A recent meta-analysis found that the response of rice yield to warming was closely related to rice variety and N rate [26]. These imply that the impact of warming on rice growth is not always consistent and varies with specific planting conditions (e.g., background temperature, variety type, management measures).

Also, we found that soil pH, AN, as well as C/N ratio were the main drivers in response to grain yield (Figure 2). On one hand, these changes reflect the treatment effects of N fertilizer on soil physiochemical properties (Table 2), a common result that chemical N fertilizer can increase the seasonal soil nutrient availability but not the TN because of the limited residue of N fertilizer [27,28]. These may further provide positive feedback to crop growth. On the other hand, the results indicated that warming tended to increase the soil pH, LOC and AN contents duo to the input and decomposition of dead ineffective tiller residue [29,30]. Generally, higher soil pH in acidic soil is beneficial in increasing the availability of electron acceptors and nutrient cycling, thus enhancing crop production [31,32]. The increased LOC and AN contents can also further increase the soil nutrient supply capacity [33]. Rehmani et al. have proved that the lower photosynthate transport during the grain filling stage is the main reason for the decrease in rice yield under warming [34]. Hence, the improvement of photosynthate transport with trialed soil nutrient supply may be the main reason for the similar yields between ET and AC. This indicates that increasing soil nutrient supply in other regions with reduced crop yields under warming may be an effective way to ensure crop production.

### 4.2. CH_4_ Emissions

Consistent with most studies, paddy CH_4_ emissions were mainly concentrated in the early growing period prior to midseason aeration (Figure 1). The emitting pattern highlights the critical role of soil moisture and the related soil EH (soil redox potential) in paddy CH_4_ emissions [35,36,37]. Generally, the variety of soil EH is a key factor affecting methane production in flooded paddy soils with ~−150 mV as the cutoff value [38]. After flooding, the soil Eh decreased sharply to ~150 mV in 10–21 days in paddies [9]. This is consistent with our emission dynamics; that is, the emission rate increased after transplanting, peaked at ~17 day and decreased rapidly after mid-season drainage (~30 d). Also, we found that the combined effect of fertilization and warming on CH_4_ emissions was not significant and the values under CN treatment increased by 79.92%. The results also indicated the grain yields were positively related to seasonal methane emissions (*p* < 0.01) and soil AN, TN, LOC and SOM were the key factors affecting CH_4_ emissions (Figure 2).

As mentioned above, N fertilizer plays a critical role in promoting crop growth and enhancing soil nutrient contents [24,27]. Generally, the variety of crop growth is accompanied by changes in root oxygen secretion, root exudates as well as litterfall [10]. On one hand, the increased root exudates, residue and crop litterfall with fertilization can be preferentially utilized by soil microorganisms; this increases soil LOC and/or promotes methane production [9,39]. On the other hand, the increased root oxygen secretion can facilitate methane oxidation in the root zone [40,41]. Combined with the above analysis, the increased methanogenic substrates resulting from the crop growth and soil nutrients are the key for the increase in paddy CH_4_ emissions under fertilization. Notably, we also found that the CH_4_ emission intensity between CN and CK treatments remained similar due to the “co-line” relationship between CH_4_ emissions and rice yield. Based on this, it is an urgent issue to establish a reasonable index to ensure the “production-CH_4_ emission” benefit of fertilization.

We also found the seasonal CH_4_ emissions with ET increased by 36.93% compared to the ambient control. Additionally, warming had a tendency to heighten the soil SOM, LOC, TN, as well as AN contents, although the differences between ET and AC were insignificant. Wang et al. pointed out that warming remarkably promoted plant growth during the vegetative growth stage, which could promote the C input [6]. Here, we also found that warming could increase the ineffective tillers at the early growth stage, although it had no significant effect on rice yield (Table 1). Thus, the input and decomposition of dead ineffective tiller residue may be the main reason for the increases in soil C (SOM, LOC) and N (TN, AN) contents. Moreover, a previous study proved that warming can accelerate the decomposition of crop residues, specifically for the non-recalcitrant C component [42]. This reveals that the increased residue input and higher decomposition rate under warming may be the principal causes of increased CH_4_ emissions.

It is well known that the activities of soil methanogens and methanotrophs play critical roles in CH_4_ emissions [9]. Surprisingly, we found that the microbial abundance related to CH_4_ emissions has an insignificant impact on paddy CH_4_ emissions, although the copies of *mcr*A and *pmo*A genes and *mcr*A/*pmo*A showed a definite response to warming (Table 3). The results highlight that it is soil substrates such as carbon sources, rather than microorganisms, that are the key factors affecting methane emissions under warming. In a green manure amendment experiment, Hou et al. also highlighted the decisive role of soil LOC in paddy methane emissions [43]. In another study, Deng et al. pointed out that the components of LOC (e.g., MBC, DOC) showed different responses to warming [30]. Hence, considering the critical role of soil LOC in paddy methane emissions, the response of SOC fractions to warming deserves attention in future research.

## 5. Conclusions

Our results showed that the rice yield was notably affected by fertilization, but not warming. The cumulative seasonal CH_4_ emissions were clearly promoted by fertilization and warming. This reveals that fertilization can aggravate the increasing effect of warming on methane emissions. Also, we found that it was soil nutrient contents (soil AN, TN, LOC and SOM) rather than microorganisms that were the key factors affecting methane emissions. It is a daunting task for researchers to optimize fertilization to ensure yield and reduce methane emissions amid global warming.

## Figures and Tables

**Table 1:** The average CH_4_ emitting rate, seasonal emissions, rice grain yield and CH_4_ emission intensity.

Treatment	Average CH_4_ Emitting Rate (mg m^−2^ h^−1^)	Seasonal CH_4_ Emissions (kg ha^−1^)	Grain Yield (t ha^−1^)	CH_4_ Emission Intensity (g kg^−1^)
AC-CN	5.67 b	122.86 b	10.65 a	11.54 a
ET-CN	8.24 a	178.08 a	11.33 a	15.72 a
AC-CK	3.70 b	74.76 b	6.16 b	12.12 a
ET-CK	4.24 b	92.51 b	6.32 b	14.64 a
*F* values				
Fertilization (F)	19.52 **	21.95 **	19.87 **	ns
Temperature (T)	5.33 *	6.54 *	ns	3.54 †
F × T	ns	ns	ns	ns

Note: AC, ambient control; ET, experimental warming; CN, fertilization treatment; CK, no N-fertilizer input treatment. Values followed by different lowercase letters indicate significant difference (*p* < 0.05). ns, not significant; † *p* < 0.1; * *p* < 0.05; ** *p* < 0.01.

**Table 2:** Soil physicochemical properties as affected by temperature and fertilization.

Treatment	pH	SOM (g kg^−1^)	TN (g kg^−1^)	C/N	LOC (g kg^−1^)	AN (mg kg^−1^)
AC-CN	5.52 b	29.28 a	1.97 a	8.62 a	1.96 ab	203.17 a
ET-CN	5.87 a	30.90 a	2.08 a	8.64 a	2.18 a	207.67 a
AC-CK	5.94 a	27.67 a	1.81 a	8.86 a	1.75 b	178.67 a
ET-CK	6.02 a	30.22 a	2.01 a	8.74 a	1.93 ab	199.33 a
*F* values						
Fertilization (F)	8.42 *	ns	ns	ns	4.79 †	3.66 †
Temperature (T)	4.94 †	ns	ns	ns	3.74 †	ns
F × T	ns	ns	ns	ns	ns	ns

Note: Values followed by different lowercase letters indicate significant difference (*p* < 0.05). ns, not significant; † *p* < 0.1; * *p* < 0.05.

**Table 3:** *F* values for the effects of temperature and fertilization on the copies of *mcr*A, *pmo*A genes and *mcr*A/*pmo*A ratio.

Variables	Fertilization (F)	Temperature (T)	F × T
*mcr*A	ns	14.17 **	ns
*pmo*A	ns	8.15 *	ns
*mcr*A/*pmo*A	ns	4.37 †	ns

Note: ns, not significant; † *p* < 0.1; * *p* < 0.05; ** *p* < 0.01.

## Data Availability

The datasets generated and analyzed during the current study are available from the corresponding author on reasonable request.
